# Beyond Single Behaviors: A Systems Perspective on Physical Activity, Nutrition, and Lifestyle in Children’s Health

**DOI:** 10.3390/nu18091394

**Published:** 2026-04-29

**Authors:** Agnieszka Kozioł-Kozakowska, Małgorzata Wójcik

**Affiliations:** 1 Department of Pediatrics, Gastroenterology and Nutrition, Institute of Pediatrics, Faculty of Medicine, Medical College, Jagiellonian University, Wielicka 265 St., 30-663 Kraków, Poland; 2 Interclinical Center for the Treatment of Childhood Obesity, University Children’s Hospital in Kraków, Wielicka 265 St., 30-663 Kraków, Poland; malgorzata.wojcik@uj.edu.pl; 3 Department of Pediatric and Adolescent Endocrinology, Chair of Pediatrics, Institute of Pediatrics, Faculty of Medicine, Medical College, Jagiellonian University, Wielicka 265 St., 30-663 Kraków, Poland

The health of children and adolescents is increasingly understood as the result of complex interactions between biological predispositions and modifiable lifestyle factors, particularly dietary intake, physical activity, and psychosocial environment. Global analyses confirm a dramatic increase in the prevalence of overweight and obesity among children and adolescents over recent decades, reflecting a sustained shift in population health trajectories [1]. At the same time, insufficient physical activity remains highly prevalent, with more than 80% of adolescents failing to meet recommended levels worldwide [2]. This persistent gap between knowledge and implementation highlights a critical limitation of current prevention strategies: their insufficient capacity to address the complexity and interdependence of lifestyle determinants.

A key concept emerging from contemporary research is the early establishment and persistence of health trajectories. Longitudinal evidence demonstrates that body mass index (BMI) and related metabolic patterns track from childhood into adulthood, indicating that early deviations from healthy growth trajectories are likely to persist without intervention [3]. This has profound implications for clinical practice and public health, emphasizing the need to prioritize early-life interventions and continuous monitoring rather than delayed, reactive strategies.

However, focusing solely on anthropometric indicators risks oversimplifying the problem. Dietary patterns among children increasingly reflect high consumption of ultra-processed foods, which are associated with poor diet quality, excessive energy intake, and adverse metabolic outcomes [4]. Importantly, such dietary behaviors are embedded within broader socio-economic and environmental contexts, including family dynamics, food systems, and cultural norms. This reinforces the need for systemic, rather than purely individual-level, interventions.

Physical activity remains a cornerstone of healthy development, contributing to bone health, cardiometabolic function, and mental well-being [2]. Nevertheless, its isolated impact on obesity prevention is often overestimated. Evidence indicates that physical activity alone produces relatively modest effects on body weight when not combined with dietary modification, although it significantly improves other health outcomes [5]. Furthermore, even physically active children may present with suboptimal nutritional habits or health-related complaints, underscoring the limitations of single-component approaches.

The effectiveness of combined lifestyle interventions is well established. Interventions integrating dietary modification and physical activity demonstrate synergistic benefits across multiple health domains, including body composition and metabolic health [5]. However, a consistent challenge lies in translating short-term behavioral improvements into sustained reductions in obesity prevalence. Many interventions show limited impact on anthropometric outcomes, particularly when implemented over short durations or within a single setting.

This limitation highlights the importance of adopting long-term, multi-level strategies that extend beyond individual behavior change. Evidence suggests that interventions are more effective when they simultaneously engage families, schools, healthcare systems, and communities, thereby addressing the broader determinants of health [5]. Additionally, socio-economic inequalities must be considered, as they significantly influence both exposure to risk factors and access to effective interventions.

The psychosocial dimension of pediatric health represents another critical, yet often underestimated, factor. Weight-related stigma remains widespread and is strongly associated with adverse mental health outcomes, including depression, anxiety, and disordered eating behaviors [6]. Importantly, stigma can undermine treatment engagement and reduce the effectiveness of interventions, highlighting the need for supportive, non-stigmatizing approaches in both clinical and public health settings.

Emerging research further suggests that dietary exposures may influence endocrine and developmental processes, including the timing of puberty. While evidence remains evolving, associations between childhood obesity, nutritional factors, and earlier pubertal onset have been increasingly reported, suggesting a broader role of nutrition in developmental regulation [7].

Taken together, the contributions to this Special Issue support a shift from reductionist approaches toward integrated, systems-based strategies. Children’s health is not determined by isolated behaviors but by networks of interacting factors operating across biological, behavioral, and environmental domains.

## Conclusions

The evidence underscores the need to move beyond fragmented, short-term interventions toward comprehensive, sustained strategies that reflect the multifactorial nature of pediatric health. Effective prevention and management require early-life intervention, integration of dietary and physical activity components, attention to psychosocial determinants, and coordinated action across multiple levels.

Future research should prioritize longitudinal designs, standardized methodologies, and the development of scalable, equitable interventions capable of producing sustained health benefits across diverse populations.
